# Binding of human serum proteins to *Plasmodium falciparum*-infected erythrocytes and its association with malaria clinical presentation

**DOI:** 10.1186/s12936-020-03438-8

**Published:** 2020-10-08

**Authors:** Mary Lopez-Perez, William van der Puije, Filip C. Castberg, Michael F. Ofori, Lars Hviid

**Affiliations:** 1 Centre for Medical Parasitology, Department of Immunology and Microbiology, Faculty of Health and Medical Sciences, University of Copenhagen, Copenhagen, Denmark; 2 Department of Immunology, Noguchi Memorial Institute for Medical Research, University of Ghana, Legon, Ghana; 3 West African Centre for Cell Biology of Infectious Pathogens (WACCBIP), College of Basic and Applied Sciences, University of Ghana, Legon, Ghana; 4 Centre for Medical Parasitology, Department of Infectious Diseases and Department of Clinical Microbiology, Rigshospitalet, Copenhagen, Denmark

**Keywords:** α_2_-Macroglobulin, Ghana, Malaria, Non-specific IgM, PfEMP1, *Plasmodium falciparum*, Rosetting, Severe malaria

## Abstract

**Background:**

The pathogenesis of *Plasmodium falciparum* malaria is related to the ability of parasite‑infected erythrocytes (IEs) to adhere to the vascular endothelium (cytoadhesion/sequestration) or to surrounding uninfected erythrocytes (rosetting). Both processes are mediated by the expression of members of the clonally variant PfEMP1 parasite protein family on the surface of the IEs. Recent evidence obtained with laboratory-adapted clones indicates that *P. falciparum* can exploit human serum factors, such as IgM and α_2_-macroglobulin (α_2_M), to increase the avidity of PfEMP1-mediated binding to erythrocyte receptors, as well as to evade host PfEMP1-specific immune responses. It has remained unclear whether PfEMP1 variants present in field isolates share these characteristics, and whether they are associated with clinical malaria severity. These issues were investigated here.

**Methods:**

Children 1–12 years reporting with *P. falciparum* malaria to Hohoe Municipal Hospital, Ghana were enrolled in the study. Parasites from children with uncomplicated (UM) and severe malaria (SM) were collected. Binding of α_2_M and IgM from non-immune individuals to erythrocytes infected by *P. falciparum* isolates from 34 children (UM and SM) were analysed by flow cytometry. Rosetting in the presence of IgM or α_2_M was also evaluated. Experimental results were analysed according to the clinical presentation of the patients.

**Results:**

Clinical data from 108 children classified as UM (n = 54) and SM cases (n = 54) were analysed. Prostration, severe malaria anaemia, and hyperparasitaemia were the most frequent complications. Three children were diagnosed with cerebral malaria, and one child died. Parasite isolates from UM (n = 14) and SM (n = 20) children were analysed. Most of the field isolates bound non-immune IgM (33/34), whereas the α_2_M-binding was less common (23/34). Binding of both non-immune IgM and α_2_M was higher but not significant in IEs from children with SM than from children with UM. In combination, IgM and α_2_M supported rosette formation at levels similar to that observed in the presence of 10% human serum.

**Conclusions:**

The results support the hypothesis that binding of non-immune IgM and/or α_2_M to IEs facilitates rosette formation and perhaps contributes to *P. falciparum* malaria severity.

## Background

Malaria continues to be an important public health problem in the developing world. Despite intensive global efforts, the number of malaria cases worldwide has not changed for the past 4 years. In 2018, an estimated 228 million cases of malaria and 405,000 deaths occurred worldwide, most of them in Africa. Notably, 67% of global deaths occurred in children aged under 5 years [1]. The broad spectrum of malaria-related manifestations ranges from asymptomatic parasitaemia to severe, life-threatening disease. Of the species infecting humans, *Plasmodium falciparum* is responsible for the vast majority of clinical cases as well as for almost all severe morbidity and mortality [1].

The pathogenesis of *P. falciparum* malaria is in part related to adhesion of parasite-infected erythrocytes (IEs) to the vascular endothelium (cytoadhesion/sequestration) in various tissues [2, 3] or to surrounding, uninfected erythrocytes (rosetting) [4, 5]. Sequestration prevents the IEs destruction in the spleen [6], but can cause tissue inflammation and organ-specific complications [7, 8]. In both, cytoadhesion and rosetting, the IEs bind to host cell membrane receptors via a diverse family of parasite-encoded protein ligands called *P. falciparum* erythrocyte membrane protein 1 (PfEMP1) [9, 10]. These proteins are displayed on multi-protein complexes (“knobs”) protruding from the IE surface [11]. Each parasite genome contains ~ 60 PfEMP1-encoding *var* genes [12, 13], but only a single PfEMP1 variant at a time is expressed on the surface of a given IE due to allelic exclusion [14]. However, the parasites can switch transcription among the different *var* genes to evade acquired PfEMP1-specific immunity. The set of *var* genes varies substantially among *P. falciparum* genomes, creating a vast global repertoire of PfEMP1 proteins. PfEMP1 variants mediating formation of rosettes also bind to endothelial cells via distinct receptor–ligand interactions [15]. The receptor specificity is facilitated by the PfEMP1 secondary structure, with defined domains mediating distinct cytoadhesion phenotypes, which in turn have been associated with discrete clinical presentations [9]. This family of proteins mediate IE adhesion to a range of host receptors, including CD36 [16], intercellular adhesion molecule 1 (ICAM-1) [17, 18], endothelial protein C receptor (EPCR) [18, 19], gC1qR [20], and oncofetal chondroitin sulfate (a.k.a. CSA) [21].

PfEMP1 also binds soluble plasma factors. Several PfEMP1 variants can bind IgM via the Fcµ region of the antibody rather than by the hypervariable, antigen-specific Fab fragment [22–24]. This type of IgM binding is sometimes called “non-immune”. Recently, was documented that non-immune IgM-binding PfEMP1 proteins are frequent in *P. falciparum* laboratory clones [24, 25], and that α_2_-macroglobulin (α_2_M), another abundant serum protein, also binds to PfEMP1 [26]. Whether PfEMP1 variants present in field isolates share these binding characteristics, and whether those features are associated with the clinical presentation of malaria are unknown. Therefore, the non-immune IgM and α_2_M binding was analysed in parasites from Ghanaian children with uncomplicated and severe malaria.

## Methods

### Ethical statement

The study was approved by the Noguchi Memorial Institute for Medical Research Institutional Review Board (NMIMR STC Number: STC Paper 5(1) 2013–2014) and by the Ethical Review Committee of the Ghana Health Service (026/13–14). Declaration of free willingness to participate in the study and written informed consent was obtained from parents/guardians of all study participants prior to enrolment.

### Study area

This study was carried out within the frame of a broader study aimed at building malaria vaccine research capacity in Ghana (MAVARECA https://mavareca.ku.dk/). The participants were enrolled in Hohoe, a town located in the Volta Region about 220 km northeast of Accra. Malaria transmission intensity in the area is high with approximately 65 infectious bites per person per year and has two seasonal peaks, a major one in April–July and a minor one in September–November [27].

### Study participants and laboratory tests

Children 1–12 years of age and reporting with *P. falciparum* malaria to Hohoe Municipal Hospital were enrolled in the study between July–August 2016. A sample size of 96 children was calculated based on 40,092 malaria cases confirmed at the Hohoe Municipal hospital in 2014 [28], using a level of confidence of 95%, sampling error of 10%, and 50% expected prevalence for IgM or α_2_M binding. After enrolment, a project nurse and a physician completed a standardized questionnaire and performed a clinical examination. Severe malaria (SM) was defined according to the WHO criteria [29], and children were treated with artemether–lumefantrine or quinine IV as required. Venous blood samples were taken on the day of admission to determine haemoglobin levels (Hb), ABO blood group, and for research purposes. Samples were taken daily during the hospitalization and 1 week after initial presentation to assess haemoglobin levels and parasitaemia. Sickle cell Hb phenotype was determined by electrophoresis and glucose-6-phosphate dehydrogenase (G6PDH) deficiency tested by methylene blue reduction test [30].

### Field isolates and in vitro culture

After removal of plasma, the pellet containing IEs was washed twice in RPMI 1640 medium (Sigma-Aldrich, Germany) supplemented with 50 µg/mL gentamicin (Sigma-Aldrich, Germany). A 100 µL aliquot of the pellet was placed in RPMI 1640 medium supplemented with 0.5% AlbuMAX II (Gibco-Life Technologies, Denmark), 2% heat-inactivated normal human serum (NHS), 2 mM glutamine (Sigma-Aldrich, Germany), and 50 µg/mL gentamicin (referred to as 2% complete culture medium) before culturing at 2% haematocrit. The parasites were incubated at 37 °C in 2% O_2_, 5% CO_2_, and 93% N_2_ atmosphere, before carrying out rosetting assays.

The rest of the washed pellet was gently mixed with glycerol freezing solution, aliquoted into cryotubes, and transferred to liquid nitrogen for long-term storage. Frozen samples were shipped to the University of Copenhagen, where they were thawed by standard methods before starting short-term in vitro cultures [31]. Briefly, the parasites were placed in 2% complete culture medium at 2% haematocrit and incubated at 37 °C in 2% O_2_, 5% CO_2_, and 93% N_2_ atmosphere. Parasitaemia was checked daily by Giemsa-stained thin smears and those with normal morphology that matured to the late-trophozoite stage were included in the study. The day before use in experiments including serum proteins, the parasites were transferred to serum-free RPMI 1640 medium (0.5% AlbuMAX II).

### Rosetting assays

The rosetting of fresh isolates was assessed in the first cycle of in vitro growth when the majority of the parasites had reached the trophozoite-late stage. After staining the parasites with 7 μg/mL of ethidium bromide for 2 min, the percentage of rosettes was assessed by counting 200 ethidium bromide-stained IEs, using wet preparations and fluorescence microscopy. Rosettes were defined as IEs having two or more adhering uninfected erythrocytes.

To determine the role of non-immune IgM and α_2_M binding in rosetting, short-term in vitro cultures of thawed cryostabilates were used. Late trophozoite stage IEs at 2% haematocrit in serum-free RPMI 1640 medium were incubated 1 h at 37 °C in 2% O_2_, 5% CO_2_, and 93% N_2_ atmosphere with 10% NHS, or with 10 nM IgM (Sigma-Aldrich, Germany), 10 nM α_2_M (Sigma-Aldrich, Germany), or both. The percentage of rosettes was assessed as described above.

### Measurements of non-immune IgM and α_2_M binding to PfEMP1

Non-immune IgM and α_2_M binding to IEs was detected by flow cytometry as previously described [24, 26, 32]. Binding of both proteins at 10 nM has been detected in several laboratory clones [23–26], but included two additional concentrations (1 nM and 100 nM) to explore the potential binding in the field isolates. Briefly, intact and unfixed late trophozoite stage IEs purified by magnet-activated cell sorting (MACS) were incubated with either 1, 10, or 100 nM non-immune human IgM or α_2_M in PBS supplemented with 1% Ig-free bovine serum albumin (PBS 1% BSA). IE-bound IgM was measured with a FITC-conjugated anti-human IgM (1:150; Sigma), whereas α_2_M was determined with a combination of goat polyclonal anti-human α_2_M (1:300; Abcam, UK) and FITC-conjugated anti-goat IgG (1:150; Vector, UK). Non-immune IgM and α_2_M binding to IEs was quantified as the median fluorescence intensity (MFI) in IEs labelled with 10 µg/mL Hoechst. BD LSR II flow cytometer was used for data acquisition and FlowLogic software (Inivai Technologies, Australia) for data analysis.

### Statistical analysis

Data were analysed and plotted using IBM SPSS Statistics for Macintosh, Version 26.0 (IBM Corp) and GraphPad Prism version 8.0 (GraphPad Software, San Diego, California, USA), respectively. Nominal variables were analysed using descriptive statistics. The Mann–Whitney U or Friedman test and Kruskal–Wallis test followed by Dunn’s multiple comparison test were used to compare two and more-than-two groups, respectively. Spearman’s rank correlation (r_s_) was used to assess the correlation between numeric variables. Fisher’s exact test was used to compare proportions. Any *p*-value < 0.05 was considered statistically significant.

Multiple linear regression models were used to evaluate the effect of potential confounders on the relationship between percentage of rosettes or protein binding (dependent variables) and relevant independent factors. Models were adjusted by clinical category (UM and SM), age, parasitaemia, haemoglobin levels at admission, ABO blood group, and days of sickness.

## Results

### Demographic and epidemiological characteristics

A total of 113 patients were recruited to participate in the study. Five patients were excluded from the analysis because incomplete records did not allow clinical classification. The analysis presented here includes 108 children classified either as UM (n = 54) or SM (n = 54). Overall, a similar proportion of male and female were enrolled (1.1 to 1), with a mean age of 5 years (median: 5 years; interquartile range (IQR): 3–7 years). Most children presented at the hospital within a few days after onset of symptoms (median: 3 days; IQR 2–4 days), although some (22%) reported after 5–22 days of sickness. Most of the children were admitted to the hospital (86%), where they stayed less than 8 days (median: 2 days; IQR: 1–3 days). The parasitaemia at admission and before anti-malarial treatment was determined in 87 children (81%). Most (71%) presented with > 10,000 parasites/μL (median: 37,367 parasites/µL; IQR: 857–95,822 parasites/µL). Anaemia (Hb < 11 g/dL) and severe anaemia (Hb < 5 g/dL) were observed in 69% and 8% of the children, respectively. On day seven post-admission, 83% of the children had anaemia, but none of them had severe anaemia. Sixteen SM children received blood transfusion.

**Fig. 1:**
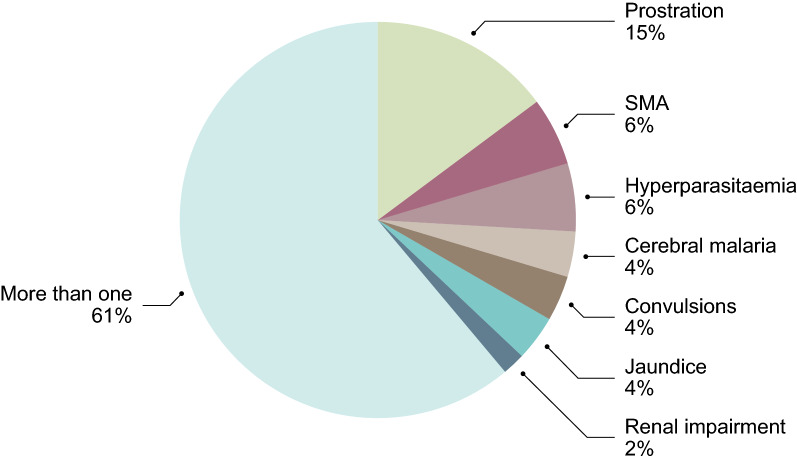
Distribution of severe malaria subcategories. Children were classified as having a single or more than one criterion. SMA: severe malaria anaemia (Hb < 5 g/dL); hyperparasitaemia (> 10%); cerebral malaria (coma with a Blantyre Coma Score  ≤ 2); multiple convulsions without coma; jaundice (plasma bilirubin > 3 mg/dL); renal impairment (creatinine > 3.0 mg/mL or urea > 20 mmol/L). More than one criterion includes children with clinical shock (< 70 mm Hg) and hypoglycaemia (< 40 mg/dL)

Some demographic and clinical variables differed between children with UM and SM (Table 1). An additional diagnosis at admission was more frequent in SM than in UM. Acute tonsillitis, gastroenteritis, and respiratory tract infections were the most frequent in both groups. Twenty-one SM children had only one criterion for classification (Fig. 1). Cerebral malaria was observed in three children; it and was the only criterion in two of them. Neither severe bleeding nor pulmonary oedema were registered. One death was reported 1 day after enrolment, in a child with clinical shock, prostration, and hyperparasitaemia.

**Table 1:** Demographic data and malaria history

	UM (n = 54)		SM (n = 54)		p value^b ^
	Median	IQR	Median	IQR	
Age (years)	6	3–8	4	2–6	*0.006*
Days of hospitalization	2	0–2	3	2–4	*< 0.0001*
Days of sickness	3	2–4	3	2–4	0.91
Parasitaemia at admission^a^	20,336	11,751–35,192	35,196	21,534–57,525	0.15
Haemoglobin at admission	10.7	10–12.2	8.0	6–10.1	*< 0.0001*

Frequencies	n	%	n	%	p value^b^
Male/female	34/20	63/37	23/31	43/57	0.05
Antimalarial preadmission	9	16.7	14	25.9	0.35
Other diagnosis at admission	17	31.5	35	64.8	*0.001*
Sickle cell Hb	2	3.7	3	5.6	1.00
G6PDH deficiency^c^	8	14.8	8	14.8	1.00

*UM* uncomplicated malaria, *SM* severe malaria, *IQR* interquartile range

^a^Geometric mean and 95% CI of geo. Mean

^b^*p* value using Mann–Whitney test or Fisher's exact test between UM and SM, significant values are highlighted in italic

^c^Full or partial deficiency

### Rosetting ex vivo

**Fig. 2:**
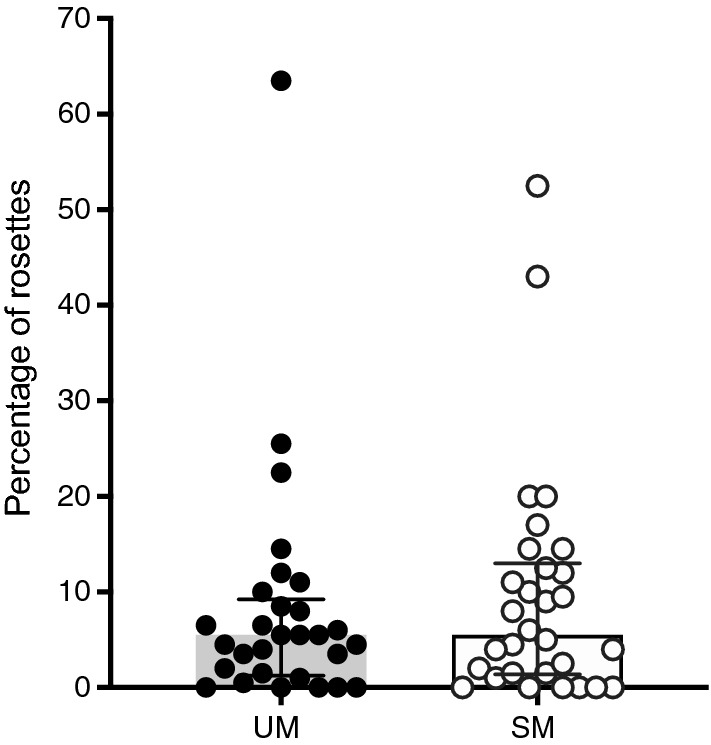
Rosetting ex vivo*.* Rosettes were defined as IEs having two or more adherent uninfected erythrocytes. Medians and interquartile ranges are shown. p = 0.64 between uncomplicated (UM) and severe malaria (SM) using Mann–Whitney test

Rosetting was assessed in 61 fresh isolates. The remaining samples (n = 47) were excluded either because the parasites failed to mature beyond ring stage in vitro or because the parasitaemia was too low for reliable assessment. Besides technical issues in the field with samples processing, a probable explanation is that children had taken anti-malarial drugs, and the parasites did not thrive overnight. Indeed, 23 out the 108 children reported taking anti-malarial drugs prior to admission (Table 1). Although most of the field isolates formed rosettes (79%), the percentage of rosettes was low (median: 7%; range 2–64%) and not different between children with UM and SM (Fig. 2). However, the percentage of rosettes was negatively correlated with age (r_s_ = − 0.373; 95% CI − 0.577 to − 0.128; p = 0.003), even after adjusting for potential confounders such as clinical category, parasitaemia, and ABO blood group (β = − 0.352; p = 0.013).

### IgM- and α_2_M-binding frequencies in UM and SM

**Fig. 3:**
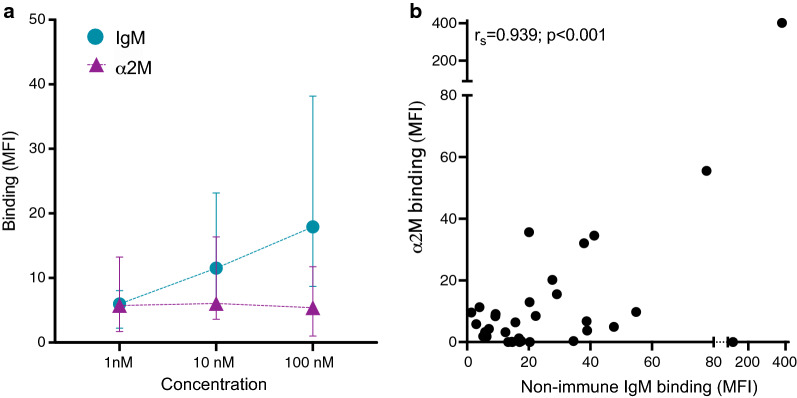
Non-immune IgM- and α_2_M-binding to *P. falciparum*-infected erythrocytes. **a **Binding of non-immune IgM (circles) and α_2_M (triangles) to IEs from 34 children. Medians and interquartile ranges are shown. **b** Correlation of non-immune IgM and α_2_M-binding at 100 nM to IEs (n = 34). Spearman’s rank correlation (r_s_) and p values are shown

Thirty-nine frozen parasite samples were thawed and put into short-term culture, with 87% successful recovery. After one to three cycles, late trophozoite stage IEs from 34 children were purified and used to evaluate binding of non-immune human IgM and α_2_M by flow cytometry. Most of the field isolates bound non-immune IgM (33/34), and the binding was dependent of the concentration (p < 0.0001; Friedman test). In contrast, the α_2_M-binding was less common than IgM (23/34; p = 0.003, Fisher’s exact test), without significant effect of increasing the concentration (p = 0.09; Friedman test) (Fig. 3a). At 100 nM, both non-immune IgM and α_2_M-binding correlated positively (r_s_ = 0.939; p < 0.001), and the association remained after adjusting for potential confounders such as clinical category, age, parasitaemia, haemoglobin levels at admission, and ABO blood group (β = 0.921; p < 0.001) (Fig. 2b).

**Fig. 4:**
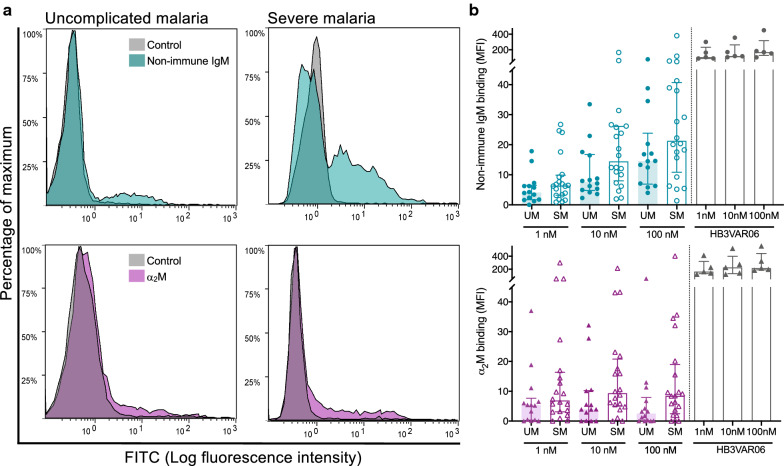
Binding of non-immune IgM and α_2_M to *P. falciparum*-infected erythrocytes. **a** Representative flow cytometry histograms of field isolates from children with uncomplicated malaria (UM) and severe malaria (SM) in the presence of non-immune IgM (above) and α_2_M (below). **b** Binding of non-immune IgM (above) and α_2_M (below) to IEs from children with UM (closed symbols, n = 14) and SM (open symbols, n = 20) at 1 nM (p = 0.24 and p = 0.15), 10 nM (p = 0.07 and p = 0.07), and 100 nM (p = 0.15 and p = 0.06), respectively. A laboratory clone, HB3VAR06, it is included for comparison. Medians and interquartile ranges are shown. p value between UM and SM using Mann–Whitney test. Two independent experiments were done at each concentration

The samples were separated into UM and SM to evaluate the association of serum protein binding with the clinical classification. At all concentrations, binding of non-immune IgM and α_2_M was higher in IEs from SM than from UM patients, although not significant at the chosen level of significance (Fig. 4). Non-immune IgM binding was negatively correlated with haemoglobin levels (r_s_ = − 0.411, p = 0.016; and r_s_ = − 0.445, p = 0.009 at 10 and 100 nM, respectively). The association was not significant after adjusting for age.

### IgM and α_2_M support rosetting in the absence of human serum

**Fig. 5:**
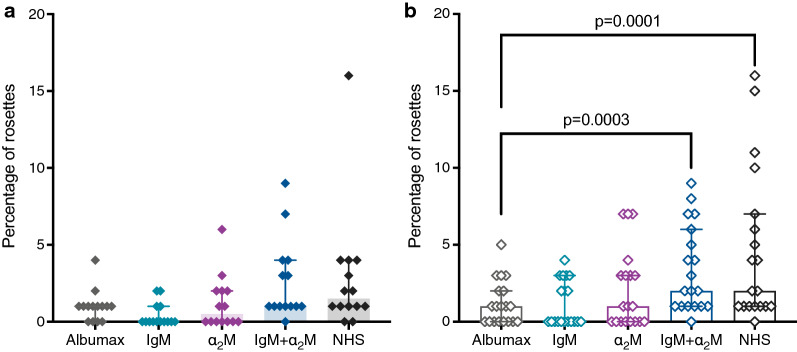
Rosetting in *P. falciparum*-infected erythrocytes. Rosetting in the absence or presence of 10 nM non-immune IgM, 10 nM α_2_M, or 10% normal human serum (NHS) in isolates from children with (**a**) uncomplicated (UM; closed symbols, n = 14) or (**b**) severe malaria (SM; open symbols, n = 20). Medians, interquartile ranges, and *p* values (Friedman test followed by Dunn’s multiple comparisons test) are shown. For each experimental condition, two independent replicates were carried out

Rosetting is a highly variable phenotype, which in many cases depends on serum factors [33]. The impact of non-immune IgM and α_2_M on rosetting was evaluated in parasite field isolates after short in vitro culture of frozen samples. Rosetting did not occur or was very low in a serum-free culture medium (0.5% AlbuMAX II), and addition of 10 nM non-immune IgM to AlbuMAX medium had no effect. In contrast, the presence of 10 nM α_2_M supported rosette formation in some isolates, and in combination, IgM and α_2_M, supported rosette formation at levels similar to that observed in the presence of 10% human serum (p = 0.86, Mann–Whitney test). Further analysis according to the clinical classification, showed that the percentage of rosettes in the presence of both IgM and α_2_M, or NHS was higher, although not significantly, in parasites from SM children than from UM children (p = 0.36 and p = 0.26, respectively, Mann–Whitney test). In field isolates from SM but not UM, the percentage of rosettes using IgM plus α_2_M or NHS was significantly higher than the observed in AlbuMAX medium (Fig. 5). The combined effect of IgM plus α_2_M in rosetting appeared additive under the conditions used here, rather than the synergistic effect previously reported for a laboratory clone [26].

## Discussion

The pathogenesis of *P. falciparum* malaria is related to the ability of the IEs to adhere to the vascular endothelium [2, 3] and to form rosettes [4, 5, 34]. Both processes are mediated by members of the PfEMP1 family proteins expressed on the IE surface [9, 10, 12, 13]. Based on evidence obtained with parasite clones adapted to long-term in vitro culture and expressing a specific PfEMP1 variant, previously was hypothesized that *P. falciparum* exploits non-immune IgM to strengthen the low-affinity interactions of PfEMP1 with the carbohydrate receptors on uninfected erythrocytes in rosetting [23]. Moreover, IgM-binding PfEMP1 proteins are common in each of the three laboratory clones studied (3D7, HB3, and IT4/FCR3) [24, 25], although not all of them mediated rosetting [24]. Similarly, α_2_M was identified as an important facilitator of rosetting that can bind at least four PfEMP1 molecules per α_2_M molecule, which might explain its effect on rosetting [26]. Remarkably, non-immune IgM and α_2_M appear to bind to the same domain in the laboratory clone HB3VAR06, where they synergistically facilitate rosetting [26].

To verify the above hypothesis, and assess its potential importance in *P. falciparum* malaria pathogenesis, the binding of non-immune IgM and α_2_M to erythrocytes infected by *P. falciparum* field isolates obtained from Ghanaian children with UM or SM was evaluated. Likewise, the rosetting in the presence of these serum proteins was tested. Using both approaches, it was found that PfEMP1 expressed on field isolate IEs can bind both non-immune IgM and α_2_M, and that both proteins allow rosetting at similar levels observed in the presence of human serum. The data suggest that binding of non-immune IgM and α_2_M is higher in SM than in UM, consistent with a role for these phenotypes in malaria pathogenesis. However, additional studies are needed to validate this hypothesis further because the differences were not statistically significant. This may be related to the fact that cerebral malaria was scarce in the study area, combined with the particular role for rosetting in CM [35, 36].

That apart, it has to be acknowledged that most of the IgM-binding PfEMP1 proteins identified to date are encoded by *var* genes belonging to group B or Group C, which are commonly found in UM and asymptomatic infections [25]. Contrary to IgM-binding, which seems to be a frequent phenotype in laboratory clones [25], α_2_M-binding is rare and restricted to DBLε and DBLζ domains (Lopez-Perez et al., unpublished data, [26]). Binding of α_2_M and non-immune IgM correlates in both field and laboratory isolates (Lopez-Perez et al., unpublished data), supporting the idea that both proteins bind to the same PfEMP1 variants. The dose–response effect observed with IgM, but not with α_2_M, it may be related to saturation. Each pentameric IgM molecule can bind two PfEMP1 molecules [23], whereas α_2_M can bind at least four [26]. The few α_2_M-binding variants to be expected among the heterogeneous PfEMP1 expressed by field isolates might easily be saturated.

The low ex vivo percentage of rosettes observed here, and the absence of the association with SM, may be related to the low number of children with cerebral malaria or severe anaemia [35, 36]. Nevertheless, the percentage of rosettes were negatively correlated with age. This is consistent with the early in life acquisition of antibodies targeting PfEMP1 variants responsible for rosetting, thus facilitating the clearance of those IEs and contributing to protective immunity to SM. Indeed, an association between higher anti-rosetting activity and age was reported in Kenyan children [37]. The differences between the field isolates tested probably reflects the high degree of PfEMP1 diversity [38], with expression of several variants within a sample collected from a single individual in contrast with a single PfEMP1 variant in most of the laboratory clones. The IgM or α_2_M levels were not measured in the NHS used for the rosetting assays. However, has been reported that α_2_M levels in IgM-depleted serum is too low to support rosetting alone, and requires the presence of IgM for rosetting to occur [26].

## Conclusions

The binding of α_2_M to IEs was uncommon, but both non-immune IgM and α_2_M-binding were slightly higher in IEs from children with SM than those with UM, although not significant at the chosen level of significance. Moreover, both proteins, α_2_M and IgM, appear to facilitate rosetting, particularly in isolates from children with SM.

## Data Availability

All data generated or analysed during this study are included within this article.

## Abbreviations

α_2_M: α_2_-Macroglobulin
Hb: Haemoglobin
IgM: Immunoglobulin M
IEs: Infected erythrocytes
NHS: Normal human serum
PfEMP1: *P. falciparum* Erythrocyte membrane protein 1
UM: Uncomplicated malaria
SM: Severe malaria
WHO: World Health Organization
